# Extracting interpretable features for pathologists using weakly supervised learning to predict p16 expression in oropharyngeal cancer

**DOI:** 10.1038/s41598-024-55288-y

**Published:** 2024-02-24

**Authors:** Masahiro Adachi, Tetsuro Taki, Naoya Sakamoto, Motohiro Kojima, Akihiko Hirao, Kazuto Matsuura, Ryuichi Hayashi, Keiji Tabuchi, Shumpei Ishikawa, Genichiro Ishii, Shingo Sakashita

**Affiliations:** 1 Department of Pathology and Clinical Laboratories, National Cancer Center Hospital East, Kashiwa, Japan; 2 Department of Otolaryngology, Head and Neck Surgery, University of Tsukuba, Tsukuba, Japan; 3 Division of Pathology, National Cancer Center Exploratory Oncology Research and Clinical Trial Center, 6-5-1, Kashiwanoha, Kashiwa, Chiba 277-8577 Japan; 4 Department of Head and Neck Surgery, National Cancer Center Hospital East, Kashiwa, Japan; 5 Department of Preventive Medicine, Graduate School of Medicine, The University of Tokyo, Tokyo, Japan; 6 Division of Innovative Pathology and Laboratory Medicine, National Cancer Center Exploratory Oncology Research and Clinical Trial Center, Kashiwa, Japan

**Keywords:** Cancer, Head and neck cancer, Diagnostic markers

## Abstract

One drawback of existing artificial intelligence (AI)-based histopathological prediction models is the lack of interpretability. The objective of this study is to extract p16-positive oropharyngeal squamous cell carcinoma (OPSCC) features in a form that can be interpreted by pathologists using AI model. We constructed a model for predicting p16 expression using a dataset of whole-slide images from 114 OPSCC biopsy cases. We used the clustering-constrained attention-based multiple-instance learning (CLAM) model, a weakly supervised learning approach. To improve performance, we incorporated tumor annotation into the model (Annot-CLAM) and achieved the mean area under the receiver operating characteristic curve of 0.905. Utilizing the image patches on which the model focused, we examined the features of model interest via histopathologic morphological analysis and cycle-consistent adversarial network (CycleGAN) image translation. The histopathologic morphological analysis evaluated the histopathological characteristics of image patches, revealing significant differences in the numbers of nuclei, the perimeters of the nuclei, and the intercellular bridges between p16-negative and p16-positive image patches. By using the CycleGAN-converted images, we confirmed that the sizes and densities of nuclei are significantly converted. This novel approach improves interpretability in histopathological morphology-based AI models and contributes to the advancement of clinically valuable histopathological morphological features.

## Introduction

Oropharyngeal squamous cell carcinoma (OPSCC) is a cancer that affects the tonsils, root of the tongue, soft palate, and uvula^1^. Human papillomavirus (HPV) infection has recently emerged as an important risk factor for OPSCC^1^. The most recent American Joint Committee on Cancer (AJCC) staging system separates HPV-positive and HPV-negative OPSCC because of their different tumor characteristics and outcomes^1,2^.

p16 overexpression, as assessed by p16 immunohistochemistry (IHC), has emerged as a surrogate marker for HPV-mediated carcinogenesis and, therefore, as a way to identify HPV-positive oropharyngeal cancer^2^. While p16 IHC is considered a sufficient standalone test for HPV status, there remains discordance between the results of p16 IHC and DNA in situ hybridization or polymerase chain reaction^3^, with discordance rates ranging from 4 to 20%^3^. For this reason, in addition to p16 IHC, the American Society of Clinical Oncology (ASCO) guidelines state that HPV-specific testing may be performed at the discretion of the pathologist and/or treating clinician^3^. Therefore, recognizing the typical histopathological features of p16-positive cases is important for pathologists.

Clinically, HPV-positive OPSCC is associated with a more favorable prognosis than HPV-negative OPSCC^1,4^. Some trials are examining the potential for de-escalating the intensity of OPSCC treatments to improve quality of life while maintaining an acceptable survival rate^1^. However, clinical trials using HPV status as a single stratification biomarker cannot show the utility of such de-escalation treatments^4^. This is because HPV-positive patients cannot be considered a single group, as the characteristics of patients and the disease can vary^5^. For this reason, further stratification of OPSCC has been proposed^6,7^.

In recent years, artificial intelligence (AI) has been applied to various types of medical images, including histopathological images, to achieve largely improved diagnostic accuracy over human assessment^8^. In the field of histopathology, deep learning algorithms have been applied for tasks such as tumor detection, grading, subtyping, and biomarker prediction^9–15^. For HPV-positive OPSCC, some AI-based approaches have been developed for HPV infection prediction and prognosis^4,16,17^. Klein et al. reported a model for predicting the association of HPV infection with OPSCC using hematoxylin eosin (HE)-stained slides and indicated the utility of their model for stratifying patient prognosis^4^. However, attempts have been made to understand the histopathologic features that are important for AI prediction, but they are not clear.

The interpretability of AI prediction models is significant in the context of AI development and model evaluation^18^. The ability to interpret the basis for the model's decisions facilitates an assessment of whether the predictions rely on clinically relevant features. Furthermore, such interpretability enables the model to uncover novel features^18^. Various methods, such as gradient class activation mapping (Grad-CAM), have been proposed to visualize regions crucial for prediction^8,18^. These existing methods do not directly approach the important feature for prediction. Cycle-consistent generative adversarial network (CycleGAN) is an approach for unpaired image-to-image translation^19^. Specifically, when converting images, CycleGAN captures the features of one image group and translates the features of another image group^20^. In histopathological images, the utility of CycleGAN has been reported for stain normalization and stain transformation to special stains^20–22^. However, the effectiveness of CycleGAN in enhancing the interpretability of the AI model by employing it for the visualization of features on which the AI model focused has not been evaluated.

One problem with existing deep learning algorithms for making predictions from histopathological images is the heterogeneity of the tissue contained in a single slide^13^. That is, a single tissue slide usually contains an abundance of nontumor tissue in addition to tumor tissue, which dilutes the overall information content^13^. Two main approaches are available for handling the heterogeneity of tissue samples: fully supervised and weakly supervised approaches (Supplementary Table 1)^23^. Fully supervised approaches require the tumor region to be manually annotated on all slides used for training and testing, which imposes a heavy burden on the annotator^23^. In contrast, weakly supervised approaches reduce the annotation burden by labeling each slide with a single label instead of manually annotating the tumor region^11^. This type of approach also makes it possible to understand the predictive basis of the model by using images that the model focused on when making its predictions^18^. Using this predictive basis, we can confirm whether the trained model had captured reasonable features.

There is a drawback to the use of weakly supervised approaches, however. For small cancer datasets, weakly supervised approaches are uncapable of fully capturing the morphological variability of tumor tissue^24^. Indeed, the advantage of weakly supervised approaches over fully supervised approaches is that they enable training to be performed on large-scale datasets^18,24^. For example, in the weakly supervised learning of pathology images, Lu et al. reported clustering-constrained attention-based multiple-instance learning (CLAM)^9,10^. The CLAM model uses an attention mechanism to extract representative images for prediction, aiding in the interpretation of the features on which the AI model focused^15^. In one study using the CLAM model, a large-scale dataset with 22,833 slides was used for training, and the utility of the model for assessing the tumor origin for cancer of unknown origin was demonstrated^9^.

**Figure 1:**
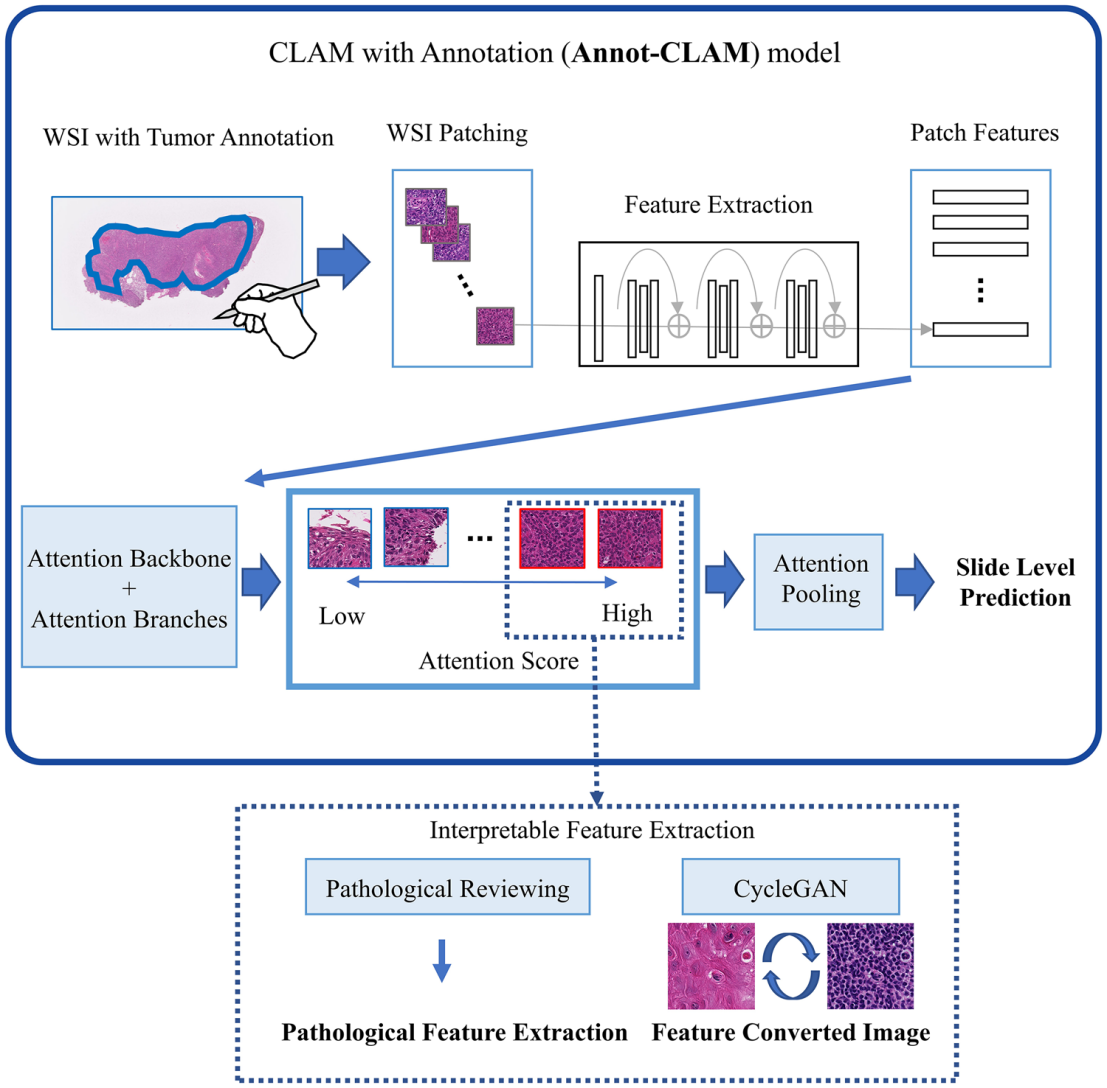
Overview of the study The Annot-CLAM model, a version of the CLAM model modified to use annotated ROIs, was applied. Two analysis approaches were used to interpret the features that the prediction model focused on.

In this study, we aimed to extract interpretable histopathologic morphological features of p16-positive OPSCC by using an AI prediction model (Fig. 1, Supplementary Fig. 1). We used the weakly supervised CLAM model for the prediction. To improve the performance of CLAM on small datasets, we modified the model to use the annotated tumor area (Annot-CLAM). Furthermore, to confirm the predictive features in a form that is easy for pathologists to understand, we attempted to visualize the features that our prediction model focused on by utilizing CycleGAN image translation. Then, to examine the reason for the discrepancy in the p16 IHC results and our model prediction results, we evaluated histopathologic morphology and gene expression using the Cancer Genome Atlas (TCGA)-Head and Neck Squamous Cell Carcinoma (HNSC) dataset. Here, we describe a novel approach for interpreting the predictive basis of weakly supervised prediction models for histopathological images. This approach introduces a new direction to utilize generative adversarial network (GAN) for model interaction.

## Results

### CLAM model performance and generated heatmaps

To predict p16 expression, we attempted to construct a model based on CLAM using the tissues of patients with OPSCC using the National Cancer Center Hospital East dataset. The dataset consisted of 116 whole-slide images (WSIs) from 114 primary OPSCC biopsy cases (50 p16-positive cases and 64 p16-negative cases). We first extracted 256 × 256 pixel patches at × 10,  × 20, and × 40 magnification levels and trained CLAM with images from each magnification level separately (Supplementary Fig. 2). We evaluated the slide-level prediction performance using tenfold cross-validation; for each fold, the performance was evaluated using a test set after the training process was completed.

**Figure 2:**
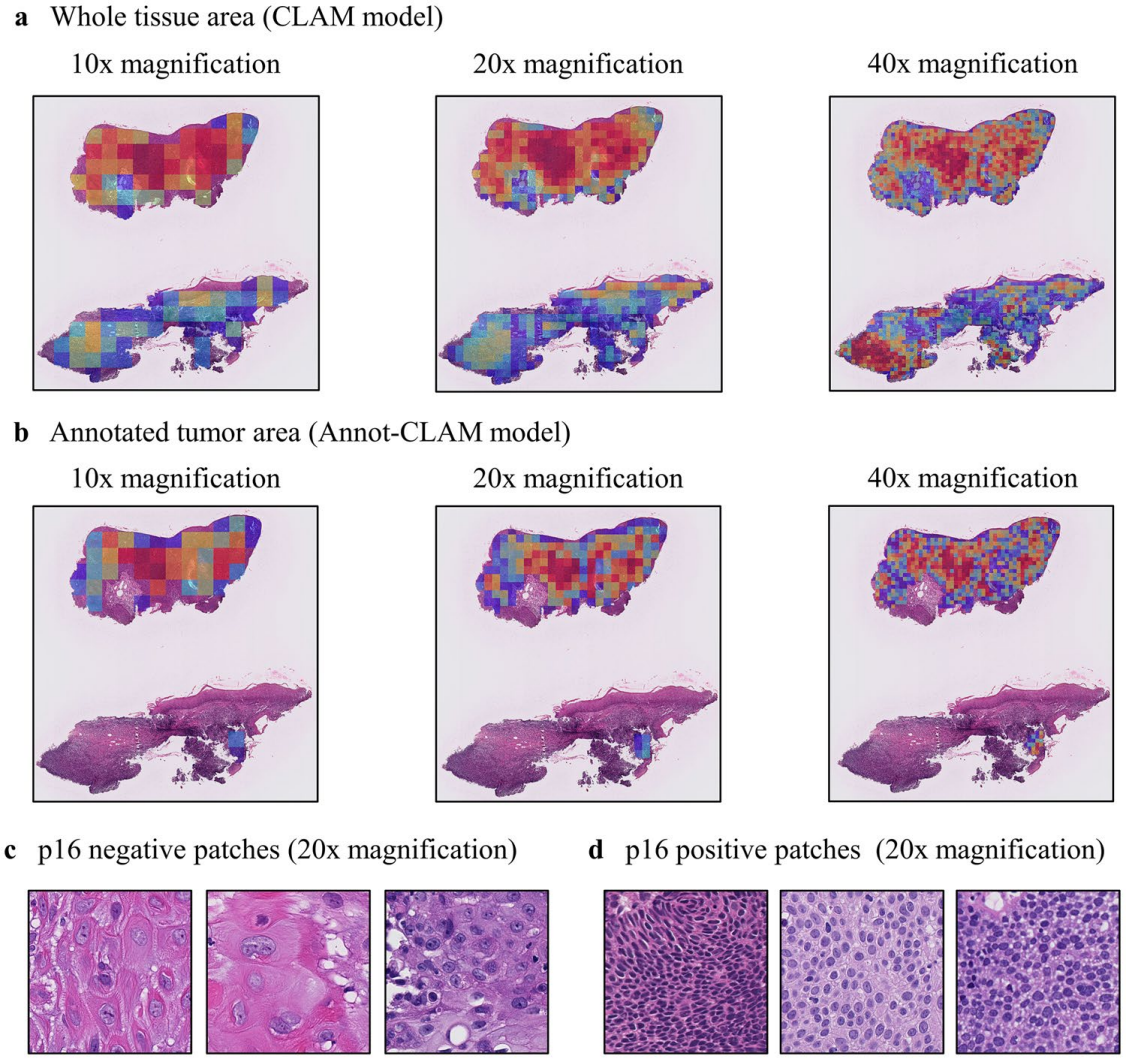
Heatmaps and highly predictive patches (a, b), Heatmaps obtained using the CLAM (**a**) and Annot-CLAM models (**b**). The heatmaps were generated by computing the attention score for the model prediction. The levels of attention given to the tumor areas by the CLAM and Annot-CLAM models differed. (**c**), Patches from p16-negative cases at 20 × magnification. The patches have low nuclear density and intercellular bridges. (**d**), Patches from p16-positive cases at 20 × magnification. The patches demonstrate high nuclear density and small nucleus size.

The mean area under the receiver operating characteristic (ROC) curve (AUC) in predicting p16 expression in OPSCC tissue ranged from 0.802 to 0.834 for CLAM models using patches with different magnifications from the whole tissue area (Table 1). The CLAM model produced interpretable heatmaps based on the contribution of each patch to the prediction process, examples of which are shown in Fig. 2a.

**Table 1:** Area under the receiver operating curve and accuracy of each model.

Magnification	Whole Tissue Area				Annotated Tumor Area			
	Best fold AUC	Mean AUC ± SD	Best fold ACC	Mean ACC ± SD	Best fold AUC	Mean AUC ± SD	Best fold ACC	Mean ACC ± SD
10x	1.000	0.802 ± 0.065	0.833	0.675 ± 0.087	1.000	0.900 ± 0.062	0.833	0.733 ± 0.062
20x	1.000	0.834 ± 0.101	0.833	0.725 ± 0.091	1.000	0.905 ± 0.072	0.917	0.775 ± 0.065
40x	1.000	0.817 ± 0.124	0.833	0.708 ± 0.100	1.000	0.900 ± 0.092	1.000	0.783 ± 0.093

ACC, accuracy; AUC, area under the receiver operating characteristic curve; SD, standard deviation.

### Annot-CLAM model performance and generated heatmaps

We assumed that the predictable patches would be mainly in the tumor cell areas. Thus, improve the performance of the model, we developed the Annot-CLAM model to use regions of interest (ROIs) in those areas. A pathologist manually annotated the tumor areas for all WSIs. The mean AUC of the Annot-CLAM models constructed with images of different magnifications using the annotated tumor areas ranged from 0.900 to 0.905, an improvement over the CLAM model results obtained using the whole tumor tissues (Table 1). The model using 20 × magnification was the best AUC. Thus, the model with magnification of 20 × was used for the rest of the study. The heatmaps indicate the differences between the levels of attention given to the tumor areas by the CLAM and Annot-CLAM models. Specifically, in the CLAM model, attention was focused on the tumor as a whole, whereas in the Annot-CLAM model, attention further stratified importance for prediction within the tumor area (Fig. 2b). Of note, the model performance improved by using the annotated tumor ROIs.

### Comparison between the Annot-CLAM model and a review performed by pathologists

Given the performance of the Annot-CLAM model described above, we next sought to compare this performance with that of pathologists in predicting p16 IHC result using HE slides. We first created a dataset containing 30 cases (15 p16-negative cases and 15 p16-positive cases) distinct from the cases used for model training and testing. The Annot-CLAM model developed with the × 20 magnification images yielded an AUC of 0.871, accuracy (ACC) of 0.800, sensitivity of 0.800, and specificity of 0.800 in this new dataset; on the other hand, the pathologist assessment of the cases yielded an average ACC of 0.692, sensitivity of 0.633 and specificity of 0.750 (Supplementary Table 2). The ACC, sensitivity, and specificity of the Annot-CLAM model were higher than those of the pathologists.

### External validation of the Annot-CLAM model

To validate our model, we used WSIs from the TCGA- HNSC project as an external and independent dataset^25^. A total of 22 oropharyngeal cancer cases used in this project underwent p16 IHC; among them, 17 yielded WSIs that were suitable for use in the model. The TCGA-datasets consisted of 12 p16-positive cases and 5 p16-negative cases. Similar to the steps performed in the comparison with the pathologists, we annotated the tumor areas of the slides and evaluated them using the Annot-CLAM model developed with the × 20 magnification images, yielding an AUC of 0.874, ACC of 0.824, and F1 score 0.889 (Supplementary Table 3). This result indicates that our Annot-CLAM prediction model can make predictions with external datasets.

### Histopathologic morphological feature analysis of highly predictive patches

The CLAM and Annot-CLAM models calculate an attention score for each patch depending on its contribution to the prediction. We extracted the highly predictive patches—those that achieved high attention scores—using the Annot-CLAM models developed with the × 20 magnification images that achieved the best performance among the models (Fig. 2c,d). From each of the test cases predicted correctly using the top three models, 5 highly predictive patches were extracted, yielding a total of 140 patches (95 p16-negative patches and 45 p16-positive patches), whose characteristics were subsequently determined. The numbers of nuclei, the perimeters of the nuclei, and the intercellular bridges of the p16-negative and p16-positive patches were significantly different (all *P* < 0.001) (Table 2).

**Table 2:** Comparison of histopathologic morphological characteristics between p16-negative and p16-positive OPSCC.

		p16-negative *n* = 95	p16-positive *n* = 45	*P* value
Mean number of nuclei, *n* (range)		68.85 (5.00–216.00)	185.13 (12.00–459.00)	< 0.001
Mean circularity of the Nuclei (range)		0.76 (0.62–0.88)	0.76 (0.40–0.88)	0.973
Mean Maximum nucleus caliper, pixels (range)		20.64 (12.83–31.79)	15.46 (10.48–21.50)	< 0.001
Mean minimum nucleus caliper, pixels (range)		12.82 (7.86–21.52)	9.69 (6.13–13.40)	< 0.001
Perinuclear halo, *n* (%)	Negative	21 (22.1)	14 (31.1)	0.297
	Positive	74 (77.9)	31 (68.9)	
Distinct nucleoli, *n* (%)	Negative	56 (58.9)	30 (66.7)	0.458
	Positive	39 (41.1)	15 (33.3)	
Intercellular bridges, *n* (%)	Negative	58 (61.1)	45 (100.0)	< 0.001
	Positive	37 (38.9)	0 (0.0)	
Keratin pearls, *n* (%)	Negative	87 (91.6)	43 (95.6)	0.501
	Positive	8 (8.4)	2 (4.4)	
Necrosis, *n* (%)	Negative	95 (100.0)	45 (100.0)	1.000

*P* values for continuous variables are based on the *t* test. *P* values for categorical variables are based on Fisher’s exact test of association.

### Feature evaluation using CycleGAN

**Figure 3:**
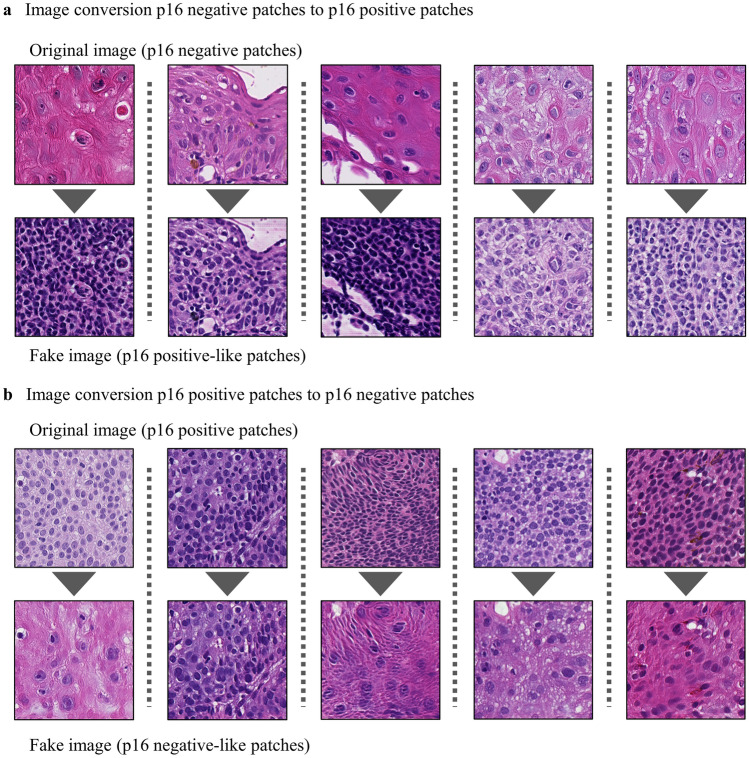
Image conversion results using CycleGAN. (**a**), Image conversion from p16-negative patches to p16-positive patches. The first row shows the original patches, and the second row shows the corresponding CycleGAN-converted patches. Small and dense nuclei were produced in the fake patches. (**b**), Image conversion from p16-positive patches to p16-negative patches. The number of nuclei decreased and the size increased in the fake patches.

Although the previous set of findings demonstrated the patch characteristics that were assessed by pathologists differed between p16-positive and p16-negative tissues, the features used by our model itself for prediction remain unknown. To interpret the features that our prediction model focused on, we evaluated the feature differences between p16-positive and p16-negative patches using CycleGAN image translation. The generator for converting between p16-positive and p16-negative patches was trained using the 1266 highly predictive patches (785 p16-negative patches and 481 p16-positive patches) extracted from the training cases predicted correctly using the top three models, and 5 highly predictive patches were extracted from each case. The feature-converted images obtained after 50 epochs of training are shown in Fig. 3. To evaluate the features changed in the patches converted using CycleGAN, we reviewed the converted features in the 140 patches used earlier for histopathologic morphological feature analysis. When p16-negative patches were converted to fake p16-positive-like patches, the mean numbers of nuclei (*P* < 0.001), the calipers of the nuclei (*P* < 0.001), the circularity of the nuclei (*P* < 0.001), the numbers of distinct nucleoli (*P* < 0.001), the intercellular bridges (*P* = 0.029), and the keratin pearls (*P* = 0.023) significantly differed. There were no significant differences in the mean numbers of nuclei, the calipers of the nuclei (*P* = 0.253), the circularity of the nuclei (*P* = 0.066), and the keratin pearls (*P* = 0.242) between the original p16-positive patches and the fake p16-positive-like patches (Table 3). When the original p16-positive patches were converted to fake p16-negative-like patches, the mean number of nuclei and the calipers of the nuclei were significantly changed (all *P* < 0.001), but these features were not significantly different between the original p16-negative patches and the fake p16-negative-like patches (Table 4). The mean number of nuclei and the calipers of the nuclei changed significantly in both directions of image conversion.

**Table 3:** Comparison of pathological characteristics following image conversion from p16-negative patches to p16-positive patches.

		p16 (−) *n* = 95	Fake p16 ( +) *n* = 95	p16 ( +) *n* = 45	*P* value p16 (−) vs. Fake p16 ( +)	*P* value p16 ( +) vs. Fake p16 ( +)
Mean number of nuclei, n (range)		68.85 (5.00–216.00)	168.91 (36.00–275.00)	185.13 (12.00–459.00)	< .001	.253
Mean circularity of nuclei (range)		0.76 (0.62–0.88)	0.78 (0.66–0.87)	0.76 (0.40–0.88)	< .001	.066
Mean maximum nucleus caliper, pixel (range)		20.64 (12.83–31.79)	15.62 (12.81–25.99)	15.46 (10.48–21.50)	< .001	.717
Mean minimum nucleus caliper, pixel (range)		12.82 (7.86–21.52)	9.61 (8.12–16.02)	9.69 (6.13–13.40)	< .001	.773
Perinuclear Halo, *n* (%)	Negative	21 (22.1)	19 (20.0)	14 (31.1)	.838	.200
	Positive	74 (77.9)	76 (80.0)	31 (68.9)		
Distinct nucleoli, *n* (%)	Negative	56 (58.9)	86 (90.5)	30 (66.7)	< .001	.001
	Positive	39 (41.1)	9 (9.5)	15 (33.3)		
Intercellular bridge, *n* (%)	Negative	58 (61.1)	69 (72.6)	45 (100.0)	.029	< .001
	Positive	37 (38.9)	26 (27.4)	0 ( 0.0)		
Keratin pearl, *n* (%)	Negative	87 (91.6)	94 (98.9)	43(95.6)	.023	.242
	Positive	8 (8.4)	1 (1.1)	2 (4.4)		
Necrosis, *n* (%)	Negative	95 (100)	95 (100)	45 (100.0)	1.00	1.00

For the comparison between p16(−) and fake p16( +) patches, *P* values were calculated using the paired *t* test for continuous variables and the McNemar test for categorical variables. For the comparison between fake p16( +) and p16( +) patches, *P* values were calculated using the *t* test for continuous variables and Fisher’s exact test for categorical variables.

**Table 4:** Comparison of pathological characteristics following image conversion from p16-positive patches to p16-negative patches.

		p16 ( +) *n* = 45	Fake p16 (−) *n* = 45	p16 (−) *n* = 95	*P* value p16 ( +) vs. Fake p16 (−)	*P* value p16 (−) vs. Fake p16 (−)
Mean number of nuclei, *n* (range)		185.13 (12.00–459.00)	56.98 (17.00–138.00)	68.85 (5.00–216.00)	< .001	.152
Mean circularity of nuclei (range)		0.76 (0.40–0.88)	0.76 (0.68–0.84)	0.76(0.62–0.88)	.882	.903
Mean maximum nucleus caliper, pixel (range)		15.46 (10.48–21.50)	21.72 (15.40–30.94)	20.64 (12.83–31.79)	< .001	.145
Mean Minimum Nucleus caliper, pixel (range)		9.69 (6.13–13.40)	13.53 (9.08–18.48)	12.82 (7.86–21.52)	< .001	.161
Perinuclear halo, *n* (%)	Negative	14 (31.1)	9 (20.0)	21 (22.1)	.302	.829
	Positive	31 (68.9)	36 (80.0)	74 (77.9)		
Distinct nucleous, *n* (%)	Negative	30 (66.7)	37 (82.2)	56 (58.9)	.146	.007
	Positive	15 (33.3)	8 (17.8)	39 (41.1)		
Intercellular bridge, n (%)	Negative	45 (100.0)	44 (97.8)	58 (61.1)	1.00	< .001
	Positive	0 ( 0.0)	1 (2.2)	37 (38.9)		
Keratin pearl, *n* (%)	Negative	43(95.6)	44 (97.8)	87 (91.6)	1.00	.271
	Positive	2 (4.4)	1 (2.2)	8 (8.4)		
Necrosis, *n* (%)	Negative	45 (100.0)	45 (100)	95 (100)	1.00	1.00

For the comparison between p16( +) and fake p16(−) patches, *P* values were calculated from the paired *t* test for continuous variables and the McNemar test for categorical variables. For the comparison between fake p16(−) and p16(−) patches, *P* values were calculated from the *t* test for continuous variables and Fisher’s exact test for categorical variables.

### Analysis of the incorrectly predicted cases using pathological review and clustering

**Figure 4:**
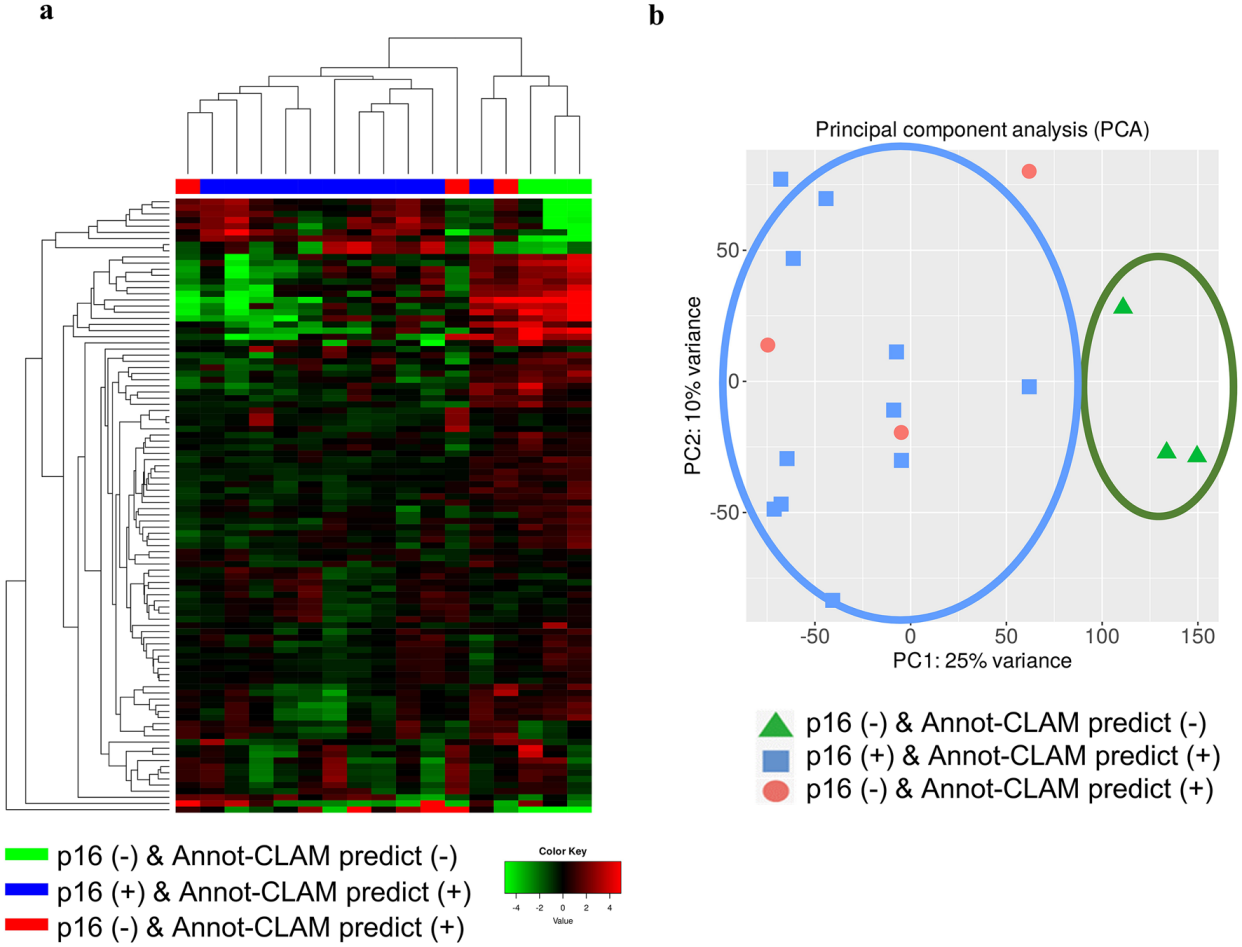
RNA sequence data from the TCGA dataset. (**a**), Hierarchical clustering and heatmaps. The top 100 variance genes were used for heatmap generation. The Euclidean distance and average variance methods were used to generate a hierarchical clustering dendrogram. (**b**), PCA plots presenting clusters of samples based on p16 IHC and Annot-CLAM prediction. p16-negative cases and those incorrectly predicted by the Annot-CLAM model were among the cluster of p16-positive cases.

The Annot-CLAM model was unable to correctly predict the labels for five cases (four p16-positive cases and one p16-negative case) in the National Cancer Center Hospital East test set. Compared to those of the correctly predicted cases, the numbers of nuclei, the perimeters of the nuclei, and the intercellular bridges of the incorrectly predicted p16-positive cases significantly differed (Supplementary Fig. 3, Supplementary Table 4). A previous report indicated the utility of the HPV prediction result using deep learning as a single biomarker with a favorable prognosis^4^. We assumed that our prediction result would correlate with prognostic factors such as gene expression. To determine the correlations between Annot-CLAM model prediction and gene expression, we performed clustering using the RNA sequence results obtained for the TCGA dataset. Three cases were found in which our model predicted false labels, all of which were p16 negative; of these, one was HPV positive. The result of hierarchical clustering is demonstrated in Fig. 4a. The p16-negative cases that the Annot-CLAM model correctly predicted were clustered into one cluster, while the p16-negative cases incorrectly predicted by the Annot-CLAM model as p16 positive were clustered separately. Principal component analysis (PCA) indicated that the cases were separated into two clusters by the Annot-CLAM model prediction method (Fig. 4b). PCA also showed that the p16-negative cases and those incorrectly predicted by the Annot-CLAM model were among the cluster of p16-positive cases. That is, the cases with a distinct histopathological morphology and which showed discrepancies between the p16 IHC results and Annot-CLAM predictions, showed different patterns of gene expression.

## Discussion

In this study, we aimed to extract features for predicting p16 expression in OPSCC tissue by using an AI model. To improve model performance, we constructed Annot-CLAM by modifying CLAM to use annotated ROIs. We interpreted the features that our prediction model focused on with two analytic approaches: a histopathologic morphological analysis of the patches with high attention scores and CycleGAN image translation analysis. Our analysis suggested that our prediction model possibly used the morphological features from histopathological images, especially the nucleus size and the number of nuclei, for p16 IHC prediction. CycleGAN could then be used to visualize these features. This visualization process is a novel and simple method for pathologists to visualize the features that the weakly supervised model focused on.

Nonkeratinizing squamous cell carcinoma has been reported as a histopathological feature of HPV-positive OPSCC^26,27^. However, it is difficult to predict HPV infections by using keratinization because only approximately 30% of HPV-positive OPSCC cases demonstrate keratinization^27,28^. In our study, the highly predictive patches indicated that the presence of an intercellular bridge, a feature of keratinization, was a significant factor in predicting p16 IHC. On the other hand, keratin pearls, another typical feature of keratinization, were not a significant factor. Our results revealed that the nuclear size and the density of the tumor cells were also important pathological features for p16 IHC prediction. Consistent with our findings, previous reports have stated that the nucleus size of HPV-related SCC is significantly smaller than that of non-HPV-related SCC, which is in accordance with the DNA ploidy of these tumors^29,30^. Overall, our results suggest that in addition to keratinization, nuclear size and density are key features for predicting p16 expression in IHC.

In this study, in addition to an attention network, we used CycleGAN to visualize the features that our prediction model focused on. Previous studies have reported methods for interpreting the reasons underlying model predictions^8,15^.Grad-CAM was reported as the method for revealing the factors that contribute to model prediction^8,31^. Grad-CAM highlights the important region for prediction, allowing CNN-based model to become more transparent by producing visual explanations^27^. The output of the important parts for prediction is different between CycleGAN and Grad-CAM, with Grad-CAM extracting important regions and CycleGAN extracting the important features themselves, not the regions. By using CycleGAN with feature translation, we confirmed the features itself extracted in the histopathologic morphological analysis. In addition, by showing the differences in features through visual information, pathologists, who routinely use visual information for diagnosis, will find it easier to recognize the differences. The use of the images produced using CycleGAN facilitates recognition of features and may improve pathologists' diagnosis; in other words, pathologists may be able to learn from the findings discovered by AI and improve their own diagnostic abilities.

From the results of our study, the histopathologic and morphologic features of incorrectly predicted p16-positive cases differed from those our model used for prediction, suggesting that there were histopathologic morphologically distinct cases. In addition, using TCGA datasets, we showed that cases with discrepancies between our predictions and the p16 IHC results had different gene expression levels from those of the correctly predicted cases. This result suggests that our histopathologic morphology-based prediction model likely focuses on features that are correlated with gene expression changes, which cannot be identified by IHC. Other studies have also investigated the relationship between histopathological morphology and molecular profile^15,32^; for example, Chen et al. reported a change in the highly predictive area when the molecular profile was combined with a WSI-based prognostic model^15^. Their findings indicated that histopathological morphology is correlated with the molecular profile, similar to the findings of our study. We believe that the advantage of AI-based histopathological evaluation lies in its ability to compare histopathological morphology with molecular features by objectively evaluating morphological features.

In this study, we employed CLAM model, a method of weakly supervised learning. Several methods of weakly supervised learning have been reported in the context of histopathological images^18,33,34^. Most weakly supervised learning models utilize methods such as multiple-instance learning and vision transformer^18^. Ghaffari Laleh et al. compared six weakly supervised learning pipelines, including the CLAM method, and reported that the accuracy of predictions varied depending on the task, with some models demonstrating superior accuracy to CLAM^34^. It is possible that other prediction models may yield more accurate results, and changes to the model may alter the important features for prediction. In many weakly supervised models, the relative importance of each image patch can be calculated^18^. Therefore, CycleGAN visualization used in this study could be applied to other weakly supervised learning models, potentially contributing to the improvement of interpretability of weakly supervised learning models for histopathological images.

This study had several limitations. First, the labels used for our model were the p16 IHC results, which, although suitable for clinical use, were in some instances incorrect, leading to false-negative and false-positive cases that affected the prediction results. For example, in the predictions obtained for the TCGA dataset, Annot-CLAM predicted positivity for p16 IHC-negative but HPV-positive cases. This means that our model was trained not on features related to p16 expression but on those related to HPV infection, which most p16-positive cases have. Labeling HPV results may therefore increase the accuracy of the model. Second, the slide images that we used were not color normalized, and therefore, we excluded features related to the color information. By using color-normalized images in the future, we may be able to improve the prediction models and identify useful color features in tissue slides.

In conclusion, we demonstrated important features for predicting p16 expression using interpretation of the AI mode. In the analysis approach using CycleGAN, features can be presented in an easily recognized form by pathologists through visualization. This is a novel approach for interpreting the predictive basis of weakly supervised prediction models for histopathological images by utilizing GAN for model interaction. This approach improves the interpretability of AI models that focus on histopathological morphology and contributes to the advancement of clinically valuable histopathological morphological features.

## Methods

### Patients and WSI datasets

We included 116 slides from 114 primary OPSCC samples for model training. These samples were obtained from patients who underwent biopsies between January 2018 and March 2021 at the National Cancer Center Hospital East. The inclusion criteria were as follows: histologically diagnosed squamous cell carcinoma from the biopsy sample and p16 IHC. The HE slides from the biopsy samples were scanned at 40 × magnification into digital slides using a NanoZoomer2.0HT digital slide scanner (Hamamatsu Photonics, Hamamatsu, Japan) and used as WSIs of this study.

p16 IHC was performed using mouse monoclonal anti-p16 antibody clone E6H4 (Roche Diagnostics, Mannheim, Germany). Subsequent steps were performed with the OptiView DAB IHC detection kit (Roche Diagnostics, Mannheim, Germany). We assessed the p16 status of each patient based on the nucleic p16 expression levels in 70% of the tumor cells in p16 IHC^2^. The p16 expression was evaluated by one pathologist and then double-checked by another pathologist. The p16 status was used for the label of this study.

The study was performed according to the Declaration of Helsinki and was approved by the institutional review board (IRB) of the National Cancer Center Hospital East (approval number 2022–142). Informed consent was waived by IRB of the National Cancer Center Hospital East. An overall flowchart of the study is presented in Fig. 1 and Supplementary Fig. 1.

### Deep learning model

To verify model performance using a weakly supervised learning approach, we implemented the CLAM model, which is easy to use and performs digital pathology on WSIs (code available at https://github.com/mahmoodlab/CLAM)^10^. The CLAM model can make predictions using a dataset of slide images and their labels. It also uses an attention mechanism to produce the images on which the model focuses when making its predictions. For this reason, this model was used in this study.

When preprocessing the slide images, CLAM segments the tissue area, which is then cropped into small patches. To create the patches, in this study, each slide is cropped into nonoverlapping 256 × 256 pixel regions from segmented images at 10x, 20x, and 40 × magnification. The training was performed separately for each magnification patch. After patch creation, CLAM extracts features from each patch by encoding into a 1024 length one-dimensional feature vector. When encoding the patches, CLAM uses a convolutional neural network (CNN) of the imagenet pretrained ResNet50 architecture. During training, the model examines and ranks all patches, assigning an attention score to each patch, which informs its contribution or importance to the collective slide-level representation for prediction^10^. The attention score is reflected in the rule of attention-based pooling for slide-level prediction, which computes the slide-level representation as the average of all patches in the slide weighted by their respective attention score^10^. During training, CLAM learns from an additional learning task of clustering the top high- and bottom low-attention scored patches into distinct clusters^10^. The total loss for a slide is calculated by summing both the slide-level prediction loss and the patch-level clustering loss^10^. For model development and evaluation purposes, a tenfold Mote Carlo cross-validation strategy was implemented in which the training/validation/testing subsets were randomly derived from the entire cohort. Specifically, for each fold, the dataset was randomly split into training (60% of cases)/validation (10%)/testing (10%) sets. Performance was assessed using the AUC and ACC. The model was trained using the adaptive moment estimation (Adam) optimizer with a learning rate of 2 × 10^–4^. We used the default algorithm for the other parameters and did not perform data augmentation^10^. The training process ended at the 200th epoch if the validation loss did not decrease from its previous minimum for 20 consecutive epochs.

### Attention heatmaps

The CLAM model calculates an attention score for each patch, allowing slide-level predictions. The attention scores are calculated by the attention branches that contribute to the prediction process and then converted between 0 and 1, with 1 being most predictive and 0 being least predictive^10^. Based on the attention scores, CLAM then produces heatmaps that allow the interpretation of the contribution of the tissue area to the model prediction process^10^. The attention scores are converted to RGB colors; patches that receive high attention scores are displayed in red (highly predictive), and patches with low attention scores are displayed in blue (less predictive). We tiled the slides into 256 × 256 pixel patches at the magnification level used for model development^10^.

### Annot-CLAM model

When training on relatively small datasets, which is an issue for weakly supervised approaches, we considered that incorporating annotations could improve the performance of the model. Therefore, we developed a CLAM model with annotations (Annot-CLAM) to extract interpretable features more efficiently. In this study, we constructed the deep learning model using the ROIs of annotations based on the CLAM model. In the segmentation and patch image generation part, we modified the CLAM model. The segmentation process was modified to use the ROI of the annotated tumor area on each slide. The tumor area was annotated by one pathologist using QuPath version 0.3.2, a publicly available annotation tool for digital slides^35^.We used the same algorithm for the parameters as the CLAM model when developing Annot-CLAM.

### Comparison of the performances of the annot-CLAM model and pathologists

We used 30 primary OPSCC biopsy cases (30 slides) from the National Cancer Center Hospital East as the dataset to compare the performance of Annot-CLAM with that of pathologists in identifying p16 expression from HE slides. The dataset consisted of 15 p16-positive cases and 15 p16-negative cases, all distinct from the cases used for model development. The HE slides were scanned into digital slides using a NanoZoomer2.0HT digital slide scanner and cropped into patches at 20 × magnification. The digital slides were assessed by the Annot-CLAM developed with 20 × magnification images with the top AUC. Eight pathology residents reviewed the slides contained in the dataset under a microscope. The analysis was performed blindly, and the assessment was based on the histopathologic morphological features of HPV-positive oropharyngeal cancer, as reported in the WHO classification^26^: (i) distinctive nonkeratinizing morphology, (ii) little surface dysplasia, (iii) growth beneath the surface epithelium lining as nests and lobules with central necrosis, (iv) tumor nests embedded in the lymphoid stroma, and (v) a high nuclear/cytoplasmic (N/C) ratio and high mitotic and/or high apoptotic ratios.

### External validation of the model

To externally validate the performance of the model with the highest AUC—the one developed with 20 × magnification images—we used the data from the public TCGA-HNSC dataset. The inclusion criteria were as follows: oropharyngeal carcinoma; available p16 IHC; and available digital histological slides. The p16 IHC data were obtained from the work published by the TCGA network^25^. WSIs of HE-stained tissue were obtained from the TCGA-HNSC dataset. Each WSI was reviewed and annotated to create the ROI of its tumor area using QuPath version 0.3.2^35^. The annotated tumor area was split into patches of 256 × 256 pixels at 20 × magnification. If the 20x-magnified image was not found in the dataset, 512 × 512 pixel patches were instead split from the 40x-magnified image and downscaled to 256 × 256 pixel patches.

### Histopathologic morphological feature analysis of highly predictive patches

To compare the histopathological features between the highly predictive patches of p16-positive and p16-negative samples, the five highly predictive patches with the highest attention scores were extracted from each of the slides. We used the slides that were correctly predicted by the three models with the highest AUCs in the test dataset used for tenfold cross-validation. These patches were reviewed by pathologists, and the features that were objectively assessable and useful for predicting the p16 IHC results were selected. The following features were systematically recorded for each patch: the number of nuclei, the circularity of the nuclei, the maximum and minimum nucleus calipers, perinuclear halos, the number of distinct nucleoli, intercellular bridges, keratin pearls, and necrosis (Supplementary Fig. 4). The number of nuclei, the circularity of the nuclei, and the maximum and minimum nucleus calipers were measured using QuPath version 0.3.2^35^. We also compared the patch features between correctly and incorrectly predicted slides.

### Feature evaluation using CycleGAN

We applied CycleGAN to translate p16-negative and p16-positive patches^19^. CycleGAN is an approach for unpaired image-to-image translation^19^. Specifically, when converting images, CycleGAN captures the features of one image group and translates them into the features of another image group^20^. CycleGAN is a GAN method that relies upon an unsupervised approach^19^. We applied CycleGAN to capture the features from the highly predictive patches of p16-positive and p16-negative samples and visualize these features by translating them. To build the training dataset for CycleGAN, we first made predictions and calculated attention scores for all slides in the dataset using the three models with the top AUC values. We then extracted the five patches with the highest attention scores from the correctly predicted slides. Duplicate extracted patches were removed to create a dataset consisting of a total of 785 p16-negative patches and 481 p16-positive patches. Finally, we applied this dataset to the CycleGAN model^19^. The model is trained for 50 epochs, and the number of epochs is chosen based on model loss and learning efficiency. We evaluated the differences between the original images and the images produced by CycleGAN using the highly predictive patches extracted from each of the correctly predicted slides in the test dataset, which were used for the pathological review discussed above. We also compared the changes in the histopathological features between the original and artificially produced images.

### RNA sequence data processing and clustering analysis

Read count data were obtained from the TCGA-HNSC dataset. We used single-stranded first RNA count data for the analysis. Normalization and analysis were performed using iDEP 0.96^36^. The read counts for all samples were normalized using EdgeR. Weakly expressed genes were excluded if they did not have more than 0.5 reads per million in at least three samples. We performed heatmap production, hierarchical clustering, and PCA using RNA count data. The top 100 variance genes were used for heatmap generation. The Euclidean distance and average variance methods were used to generate a hierarchical clustering dendrogram.

### Environmental and statistical analysis

The analysis of this study was executed on an Ubuntu 20.04 Linux system with an A100 GPU (NVIDIA, Santa Clara, CA). All statistical analyses were performed using EZR (Saitama Medical Center, Jichi Medical University, Saitama, Japan), a graphical user interface for R (The R Foundation for Statistical Computing, Vienna, Austria)^37^. A *P* value < 0.05 was considered to indicate statistical significance. We performed between-group comparisons using Fisher’s exact test and the *t* test for categorical and continuous variables, respectively. Image patches before and after CycleGAN conversion were statistically compared using the McNemar and paired *t* tests for categorical characteristics and continuous variables, respectively.

### Supplementary Information

Supplementary Information.

## Data Availability

The authors declare that the data supporting the findings of this study are available within the article and that the source data for the figures are provided with this paper. The raw patient data are under restricted access for privacy reasons. Data from the TCGA, including digital histology and RNA sequence data, are available from https://portal.gdc.cancer.gov. The HPV infection and p16 IHC results are available from the published work of The Cancer Genome Atlas Network^19^ (https://doi.org/10.1038/nature14129). All other results in support of this manuscript are available from the corresponding author upon reasonable request.

## Code availability

Our code used for the CLAM prediction and CycleGAN image translation processes is available from https://github.com/epocaipath/Annot-CLAM-CycleGAN.
