# Supplementary material for: Extracting interpretable features for pathologists using weakly supervised learning to predict p16 expression in oropharyngeal cancer
Source: Sci Rep. 2024 Feb 24;14:4506. doi: 10.1038/s41598-024-55288-y (PMC10894206; doi:10.1038/s41598-024-55288-y)

Title: Extracting interpretable features for pathologists using weakly supervised learning to predict p16 expression in oropharyngeal cancer.

Authors: Masahiro Adachi, Tetsuro Taki, Naoya Sakamoto, Motohiro Kojima, Akihiko Hirao, Kazuto Matsuura, Ryuichi Hayashi, Keiji Tabuchi, Shumpei Ishikawa, Genichiro Ishii, Shingo Sakashita

Supplementary Table 1 Comparison of fully supervised approach and weakly supervised approach

	Advantage	Disadvantage
Fully Supervised	High Performance	Pixel-level Annotation Needed<br>Lack of Interpretability
Weakly Supervised	Slide Level Label<br>(Pixel-level Annotation Not Needed)<br>Interpretability	Large Dataset Needed

Supplementary Fig.1 Flowchart of the study

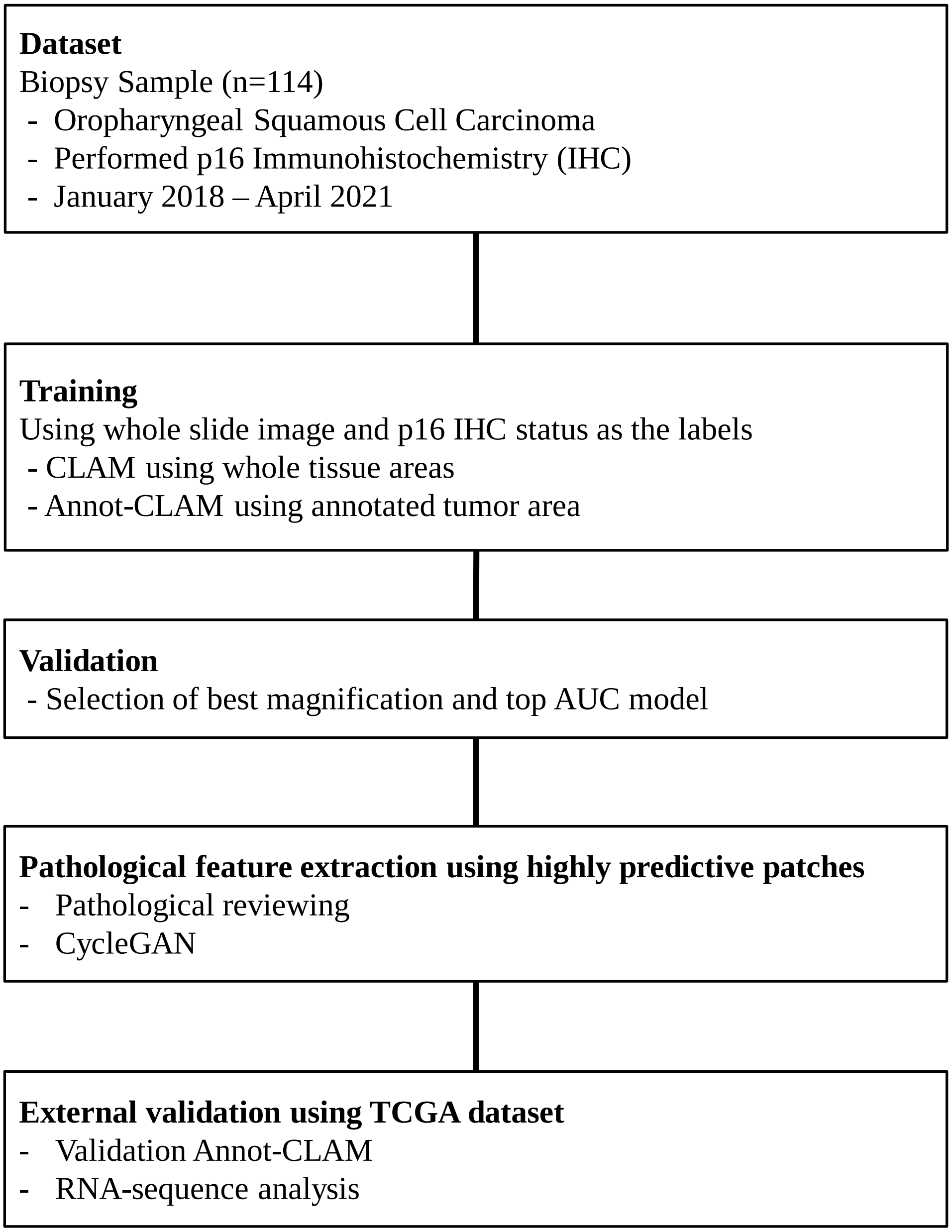

Supplementary Fig.2 Example of patches for each magnification

a p16 negative patch (256 x 256 pixel)

10x magnification (0.92μm/pixel)

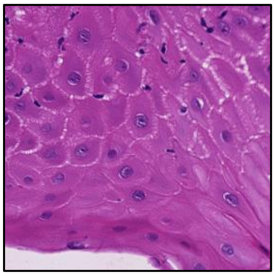

20x magnification (0.46μm/pixel)

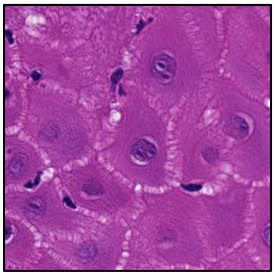

40x magnification (0.23μm/pixel)

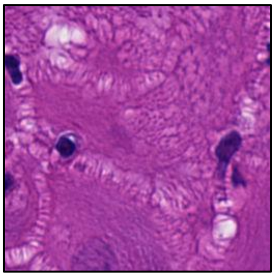

b p16 positive patch (256x256 pixel)

10x magnification (0.92μm/pixel)

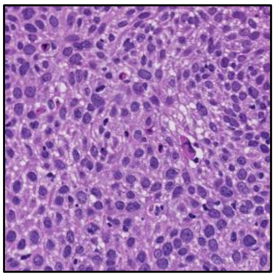

20x magnification (0.46μm/pixel)

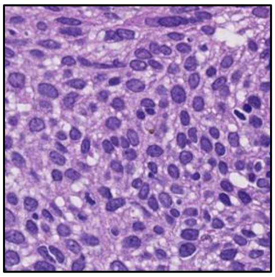

40x magnification (0.23μm/pixel)

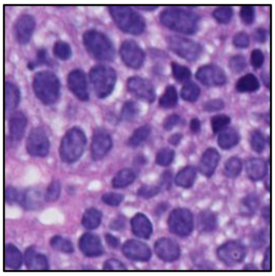

Supplementary Table 2. Comparison of Annot-CLAM model versus review of pathologists

	Annot-CLAM	Observer									
		A	B	C	D	Average	E	F	G	H	Average
Experience		board-certified pathologist					pathology residents				
Number of correct cases											
Total cases	24/30	22/30	17/30	21/30	22/30	20.5/30	20/30	25/30	17/30	22/30	21/30
p16 (-) cases	12 /15	13/15	4/15	11/15	13/15	10.25/15	11/15	13/15	10/15	15/15	12.25/15
p16(+) cases	12/15	9/15	13/15	10/15	9/15	10.25/15	9/15	12/15	7/15	7/15	8.75/15
Sensitivity	0.800	0.600	0.867	0.667	0.600	0.683	0.600	0.800	0.467	0.467	0.583
Specificity	0.800	0.867	0.267	0.733	0.867	0.683	0.733	0.867	0.667	1.000	0.817

Supplementary Table3. Validation result using cases from the TCGA-HNSC dataset

	Annotated Tumor Areas		
Magnification	AUC	ACC	F1 score
20x	0.874	0.824 (14/17cases)	0.889

ACC, Accuracy; AUC, area under the receiver operating characteristic curve

Supplementary Fig.3 Examples of highly predictive patches from incorrectly predicted case

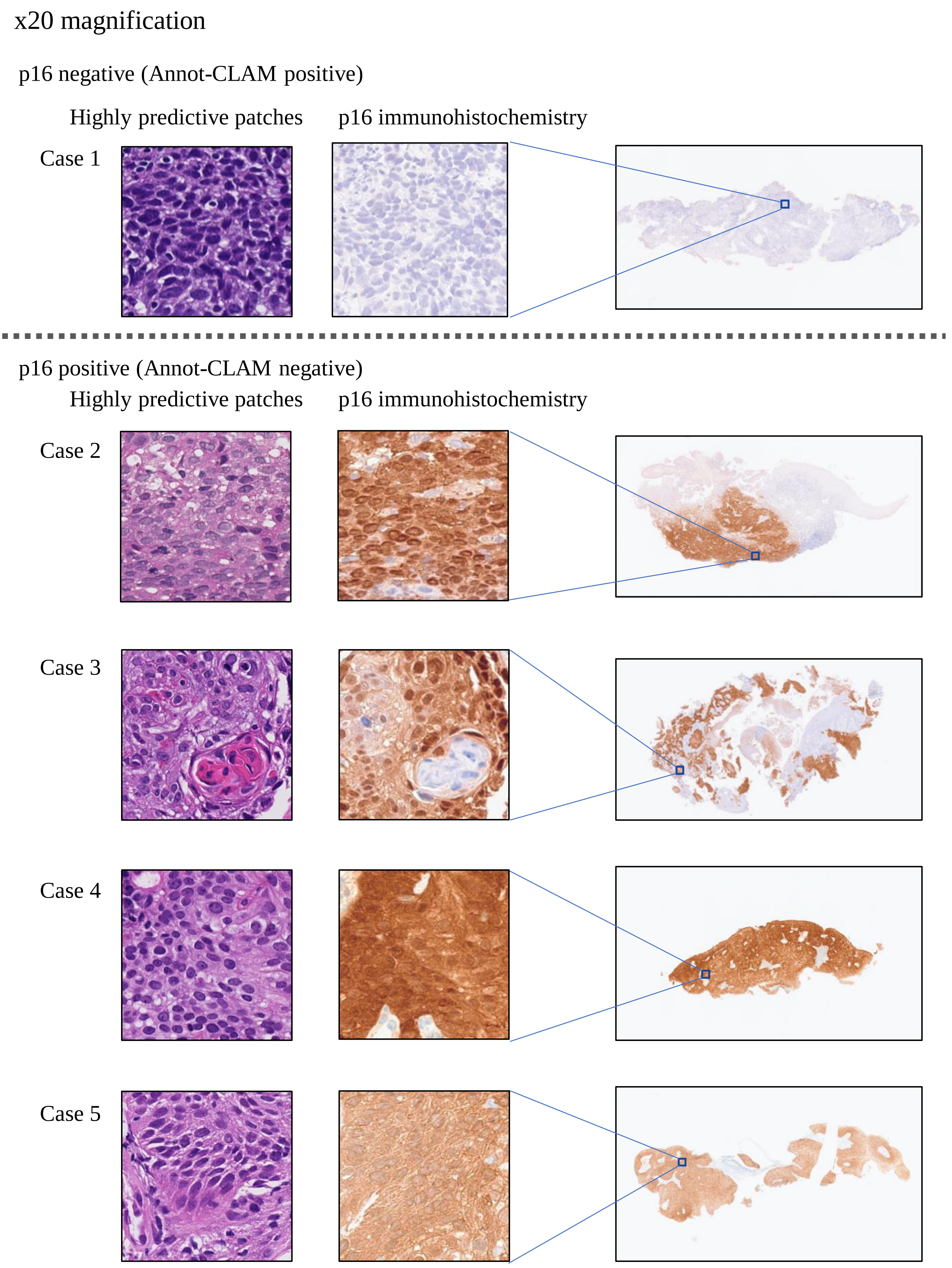

Supplementary Table 4. Comparison of pathological characteristics

Predictive patches from p16 positive and incorrectly predicted patches

		incorrect p16 (+) n=20	p16 (+) n=45	p16 (-) n=95	P-value p16 (+) vs incorrect p16(+)	P-value p16 (-) vs incorrect p16(+)
Mean Number of Nuclei, n (range)		87.20 (24.00-178.00)	185.13 (12.00-459.00)	68.85 (5.00-216.00)	.001	.124
Mean Circularity of Nuclei (range)		0.75 (0.68-0.83)	0.76 (0.40-0.88)	0.76 (0.62-0.88)	.654	.536
Mean Maximum caliper of Nuclei, pixel (range)		18.10 (14.31-22.35)	15.46 (10.48-21.50)	20.64 (12.83-31.79)	.001	.006
Mean Minimum caliper of Nuclei, pixel (range)		10.79 (7.86-12.91)	9.69 (6.13-13.40)	12.82 (7.86-21.52)	.030	.002
Perinuclear Halo, n (%)	negative	2 (10.0)	14 (31.1)	21 (22.1)	.117	.356
	positive	18 (90.0)	31 (68.9)	74 (77.9)		
Distinct Nucleous, n (%)	negative	12 (60.0)	30 (66.7)	56 (58.9)	.779	1.00
	positive	8 (40.0)	15 (33.3)	39 (41.1)		
Intercellular Bridge, n (%)	negative	17 (85.0)	45 (100.0)	58 (61.1)	.026	.068
	positive	3 (15.0)	0 (0.0)	37 (38.9)		
Keratin pearl, n (%)	negative	19 (95.0)	43(95.6)	87 (91.6)	.524	.174
	positive	1 (5.0)	2 (4.4)	8 (8.4)		
Necrosis, n (%)	negative	20 (100.0)	45 (100.0)	95 (100)	1.00	1.00

Supplementary Fig.4 Examples of pathological characteristics

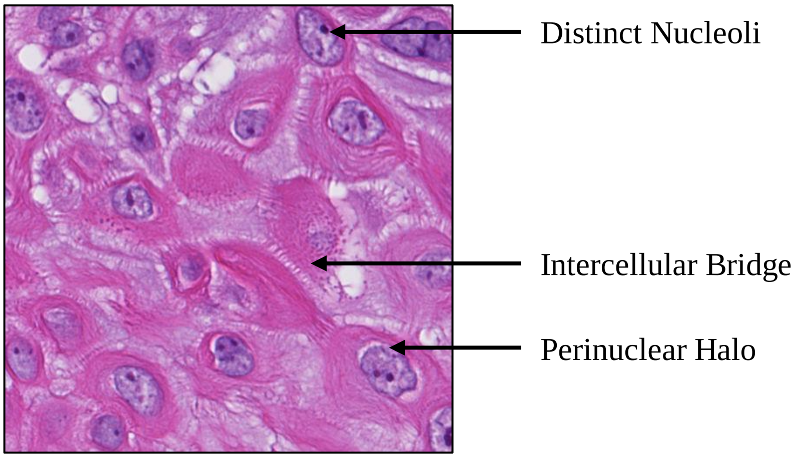

Supplement: Supplementary file 1 — Supplementary Information. [file 41598_2024_55288_MOESM1_ESM.pdf]
